# An exploratory examination of executive functioning as an outcome, moderator, and predictor in outpatient treatment for adults with anorexia nervosa

**DOI:** 10.1186/s40337-022-00602-0

**Published:** 2022-06-17

**Authors:** Ella Keegan, Susan Byrne, Phillipa Hay, Stephen Touyz, Janet Treasure, Ulrike Schmidt, Virginia V. W. McIntosh, Tracey D. Wade

**Affiliations:** 1 Discipline of Psychology, Blackbird Initiative, Órama Research Institute, Flinders University, GPO Box 2100, Adelaide, South Australia 5001 Australia; 2 SWAN Centre, Perth and School of Psychology, University of Western Australia, Perth, Australia; 3 Translational Health Research Institute, School of Medicine, Western Sydney University, Penrith, Australia; 4 InsideOut Institute, Sydney University, Sydney, Australia; 5 Department of Psychological Medicine, Kings College London, London, UK; 6 School of Psychology, Speech and Hearing, University of Canterbury Christchurch, Christchurch, New Zealand

**Keywords:** Anorexia nervosa, Executive functioning, Central coherence, Set shifting, Conceptualisation, Moderation, Outpatient treatment

## Abstract

**Objective:**

People with anorexia nervosa often exhibit inefficiencies in executive functioning (central coherence and set shifting) that may negatively impact on treatment outcomes. It is unclear from previous research whether these inefficiencies can change over treatment. We aimed to (1) investigate whether executive functioning can improve over treatment, (2) determine whether baseline executive functioning moderates treatment outcome, and (3) examine whether baseline executive functioning predicts early change (i.e., increase in body mass index over the first 13 weeks of treatment) or remission.

**Method:**

We conducted linear mixed model and logistic regression analyses on data from the Strong Without Anorexia Nervosa trial (Byrne et al. in Psychol Med 47:2823–2833, 2017). This study was a randomised controlled trial of three outpatient treatments for people with anorexia nervosa: Enhanced Cognitive Behavioural Therapy, Maudsley Model Anorexia Nervosa Treatment for Adults, and Specialist Supportive Clinical Management.

**Results:**

While set shifting clearly improved from baseline to end of treatment, the results for central coherence were less clear cut. People with low baseline central coherence had more rapid reductions in eating disorder psychopathology and clinical impairment than those with high baseline central coherence. Baseline executive functioning did not predict early change or remission.

**Discussion:**

The detail-focused thinking style commonly observed among people with anorexia nervosa may aid treatment outcomes. Future research that is more adequately powered should replicate this study and examine whether the same pattern of results is observed among people with non-underweight eating disorders.

**Supplementary Information:**

The online version contains supplementary material available at 10.1186/s40337-022-00602-0.

## Plain English Summary

People with anorexia nervosa often have difficulty thinking flexibly and in terms of the big picture. We investigated whether these thinking styles (1) change over treatment, (2) influence response to treatment, or (3) predict whether people gain weight or overcome the eating disorder. We found that people were able to think more flexibly after treatment. We also found that people who had more difficulty seeing the big picture prior to treatment had a more rapid decrease in eating disorder symptoms and clinical impairment in treatment. Thinking styles did not predict whether people gained weight early in treatment or overcame the eating disorder. Our findings suggest that the detail-focused thinking style commonly observed among people with anorexia nervosa can be both a vulnerability and a strength.

**Supplementary Information:**

The online version contains supplementary material available at 10.1186/s40337-022-00602-0.

## Background

Anorexia nervosa is a serious mental health condition that can significantly impair physical health [1], disrupt psychosocial functioning [2], and has a mortality rate six times higher than that of the general population [3]. It is also notoriously difficult to treat, with 40% of adult clients not completing stand-alone outpatient treatments, and only 28% reaching remission at 12-month follow-up [4]. Some of this poor treatment outcome may be due to the significant inefficiencies in executive functioning that are often observed among people with anorexia nervosa [5–7].

These inefficiencies in executive functioning exist in two key areas: central coherence and set shifting [5]. Inefficiencies in central coherence are characterized by excessively detail-focused thinking at the expense of the bigger picture [8], and inefficiencies in set shifting by rigid, inflexible thinking with difficulty changing responses when rules in the environment change [9]. These inefficiencies could reduce the impact of therapy for people with anorexia nervosa. For example, they could result in rigid thought patterns and behaviours related to detail about food and weight that perpetuate anorexia nervosa and could interfere with adopting a big-picture perspective about life and the need for change.

It is unclear whether these inefficiencies can change over the course of treatment. Some research has shown that central coherence and set shifting changed inconsistently or not at all over treatment [10–12]. However, other research has found that central coherence and set shifting can improve over treatment, for example, when targeted with cognitive remediation therapy (for a systematic review, see [13].

The present study utilised data from the Strong Without Anorexia Nervosa trial (SWAN; [4]. We conducted exploratory, secondary data analyses to (1) make sense of the inconsistent findings by investigating whether executive functioning can change over treatment, (2) determine whether baseline executive functioning moderates treatment outcome, and (3) examine whether baseline executive functioning predicts early change in body mass index or remission. Based on Tchanturia and colleagues [13], we predicted that executive functioning would improve over treatment. In the absence of evidence, we also predicted that people with higher executive functioning would have better outcomes, show early change, and achieve remission.

## Method

### The SWAN trial

The SWAN trial was a multi-site randomised controlled trial across three Australian states. Participants were randomly allocated to receive either Specialist Supportive Clinical Management (SSCM; n = 39), Maudsley Model Anorexia Nervosa Treatment for Adults (MANTRA; n = 41) or Enhanced Cognitive Behavioural Therapy (CBT-E; n = 40). In SSCM, the first half of sessions combines clinical management and supportive psychotherapy, whereas the second half of sessions focuses on content dictated by the client [14–16]. MANTRA targets factors maintaining anorexia nervosa, specifically, thinking style, socio-emotional impairments, close others’ unhelpful responses to the illness, and positive beliefs about anorexia nervosa [17, 18]. CBT-E for underweight clients involves motivational work, increasing dietary intake and weight, tackling eating disorder psychopathology, maintaining changes, and developing strategies to overcome setbacks [19]. Independent ratings demonstrated that all three treatments were highly distinguishable [20]. In all three treatments, participants were allocated 25 to 40 sessions based on their pre-treatment BMI (< 16 = 40 sessions; 16 ≥ 17.5 = 30 sessions; 17.5 ≤ 18.5 = 25 sessions) to allow the time required to restore weight. The trial found no significant differences in outcomes between treatments, with significant improvements across all three [4].

### Participants

The overall SWAN sample comprised 120 participants (95.8% female) who have been described previously and more fully in Byrne et al. [4]. They had a mean age of 26.19 years (*SD* = 9.47), and a mean baseline body mass index (BMI) of 16.70 kg/m^2^ (*SD* = 1.22). To be eligible for participation, they had to meet the Diagnostic and Statistical Manual of Mental Disorders (DSM-5; APA [21]) criteria for anorexia nervosa. Due to missing executive functioning data, the present study used a subset of participants from the SWAN sample (for demographic information, see Supplementary Table 1).

### Measures

Participants completed the Wisconsin Card Sorting Test (WCST; [22] and the Rey Complex Figure Test (RCFT [23, 24] at baseline and end of treatment. Treatment outcome measures were completed at baseline, mid-treatment, end of treatment, six-month follow-up, and 12-month follow-up.

#### Executive functioning

*Central coherence* The RCFT was used to assess central coherence. In this test, the participant is instructed to copy a complex figure comprising 18 numbered elements as accurately as possible. Originally, participants were instructed to produce the complex figure twice (once copying and once from memory) and both drawings were used to measure central coherence. Now, the copy trial is considered to provide the most direct measure of central coherence [25]. A central coherence index (CCI) is obtained from the order that the participant copies the first six elements of the figure (i.e., whether they copy global or detailed elements) and the style that the participant uses to copy the figure (i.e., whether they copy key global elements in a continuous or fragmented way). The CCI can range from 0 to 2, with higher scores indicating better central coherence.

*Set Shifting* Set shifting was measured using the WCST. This test requires the participant to match a number of stimulus cards to one of four key cards. Cards can be matched by the colour, form, or number of symbols on the cards. The participant is not told how to match the cards. Instead, after each trial, they are told whether they were right or wrong and they must infer the sorting rule from this feedback. Each time that the participant correctly matches 10 cards in a row, the sorting rule changes. The participant is not told that the sorting rule has changed and must infer the new sorting rule from the experimenter’s feedback. The measure of set shifting is the number of “perseverative errors” or times that the participant matches cards according to a previously correct sorting rule after the rule has changed [26]. A higher number of perseverative errors indicates poorer set shifting performance [27].

#### Treatment outcomes

*BMI* Participants’ height was measured in metres (m) at baseline. Their weight was measured in kilograms (kg) at baseline, each treatment session, and each assessment time-point. BMI was calculated as kg/m^2^.

*Eating Disorder Psychopathology *The global score of the Eating Disorder Examination (EDE; [28] was used to measure eating disorder psychopathology over the past 28 days. This semi-structured interview was administered by the Clinical co-ordinator at each site and produces four subscales: restraint, eating concern, shape concern, and weight concern. The global score is produced by summing the 22 items across the subscale scores and dividing by the number of items to obtain a mean scale score. This global score can range from 0 to 6, with higher scores indicating greater eating disorder psychopathology. The EDE has satisfactory internal consistency, discriminates well between people with eating disorders and healthy controls, and correlates with measures of similar constructs [29, 30]. In the present study, internal consistency was 0.92.

*Clinical Impairment* The 16-item Clinical Impairment Assessment (CIA; [19] was used to assess the extent of psychosocial impairment due to eating disorder psychopathology over the past 28 days. In this self-report questionnaire, items are rated from 0 to 3 and summed to produce a global score. This global score can range from 0 to 48, with higher scores indicating greater clinical impairment. The CIA has good internal consistency, discriminates between people with eating disorders and healthy controls, and is highly correlated with clinicians’ ratings of psychosocial impairment [31]. In the present study, internal consistency was 0.92.

#### Early change

Following Wade and colleagues [32], we assessed early change using change in BMI over the first 13 sessions of treatment as this represented the first “halfway point” in the 25 to 40 sessions offered in the SWAN trial. While shorter timeframes have been used in adolescents receiving Family Based Therapy (e.g., four sessions; [33]; [34], 13 sessions accounted for the less intense nature of outpatient treatment for adults with anorexia nervosa and allowed more time for changes to be observed, as has been adopted in a previous study of early change in adults [35]. Using the SWAN data, Wade and colleagues identified four latent classes with one class having a significantly greater increase in BMI over the first 13 sessions than any other class. For the purposes of the current analyses, we dichotomised early change as 0 (no early change) and 1 (early change—i.e., the people with the greatest increase over the first 13 sessions).

#### Remission

Following Byrne et al. [4], remission was defined as having a BMI greater than 18.5, a global EDE score less than 1.8, and no binge/purge behaviours.

### Statistical analyses

We conducted all analyses using IBM Statistical Package for the Social Sciences (Version 22; IBM [36]. Logistic regression analyses were conducted to determine whether baseline variables predicted missing baseline or end of treatment data for both central coherence and set shifting. We applied Bonferroni corrections for all comparisons. We conducted two linear mixed model (LMM) analyses to investigate whether executive functioning can change over treatment and whether change over treatment differed between groups. Both analyses had time and group as fixed effects and the interaction between these variables. One of these LMM analyses had central coherence as the outcome variable and the other had set shifting. To investigate whether baseline executive functioning moderated treatment outcome, we conducted a separate LMM analysis for each treatment outcome variable. These LMM analyses had fixed effects of time and baseline central coherence or set shifting and the interaction between these variables. Group was not included as a fixed effect in any of the analyses examining early change or treatment outcomes because LMM analyses require a minimum of 10 cases for each effect examined. When significant interactions were observed, we categorised participants as having low or high baseline executive functioning using a median split. Finally, to investigate whether baseline executive functioning predicted early change or remission, we conducted logistic regression analyses.

## Results

### Missing data

The pattern of missing data for each variable is described below. Both variables had a large amount of missing data at end of treatment and hence our analyses used only participants who had both baseline and end of treatment central coherence and set shifting data (as previously mentioned, demographics are provided in Additional file 1: Supplementary Table 1).

#### Central coherence

Of the 120 participants, 41 (34%) had both baseline and end of treatment data, 43 (36%) were missing both baseline and end of treatment data, 1 was missing only baseline data, and 35 were missing only end of treatment data. Baseline EDE predicted missing baseline central coherence data, such that those with higher eating disorder psychopathology at baseline were less likely to have missing baseline central coherence data. However, as shown in Table 1, no baseline variables predicted whether end of treatment central coherence data were missing. We can, therefore, conclude that end of treatment central coherence data were missing at random, and analyses contained up to 76 (63%) of the study participants.

**Table 1:** Logistic regression analyses predicting missing baseline and end of treatment executive functioning data from baseline variables

	Baseline			End of treatment		
	Missing *M* (*SD*)	Not missing *M* (*SD*)	OR (95% CI)	Missing *M* (*SD*)	Not missing *M* (*SD*)	OR (95% CI)
**Central coherence**						
Duration	8.29 (5.78)	5.93 (8.47)	0.97 (0.92, 1.02)	7.35 (9.51)	4.76 (7.41)	0.96 (0.91, 1.02)
AN subtype	AN-R: 19 (43.2) AN-BP: 25 (56.8)	AN-R: 34 (44.7) AN-BP: 42 (55.3)	0.94 (0.44, 1.99)	AN-R: 17 (48.6) AN-BP: 18 (51.4)	AN-R: 17 (41.5) AN-BP: 24 (58.5)	1.33 (0.54, 3.31)
BMI	16.80 (1.26)	16.65 (1.19)	0.90 (0.66, 1.24)	16.57 (1.27)	16.72 (1.11)	1.11 (0.76, 1.64)
EDE	2.97 (1.37)	3.52 (1.39)	1.33 (1.01, 1.74)*	3.57 (1.51)	3.48 (1.30)	0.96 (0.69, 1.33)
CIA	32.97 (9.42)	32.84 (11.45)	1.00 (0.96, 1.04)	31.35 (12.39)	34.14 (10.56)	1.02 (0.98, 1.07)
**Set shifting**						
Duration	5.75 (4.57)	6.75 (8.76)	1.02 (0.95, 1.09)	8.21 (9.31)	5.24 (8.00)	0.96 (0.90,1.02)
AN subtype	AN-R: 16 (51.6) AN-BP: 15 (48.4)	AN-R: 37 (41.6) AN-BP: 52 (58.4)	1.50 (0.66, 3.41)	AN-R: 23 (44.2) AN-BP: 29 (55.8)	AN-R: 14 (37.8) AN-BP: 23 (62.2)	1.30 (0.55, 3.08)
BMI	16.68 (1.28)	16.71 (1.19)	1.02 (0.72, 1.44)	16.66 (1.25)	16.77 (1.12)	1.08 (0.76, 1.55)
EDE	2.93 (1.47)	3.46 (1.36)	1.31 (0.97, 1.76)	3.43 (1.47)	3.49 (1.22)	1.03 (0.75, 1.41)
CIA	33.75 (10.30)	32.62 (10.94)	0.99 (0.95, 1.03)	31.80 (11.53)	33.75 (10.13)	1.02 (0.98, 1.06)

^*^Those with higher eating disorder psychopathology at baseline were less likely to have missing baseline central coherence data

For end of treatment, the analyses were only conducted for those who also had baseline data. OR = odds ratio; CI = confidence interval; AN = anorexia nervosa; AN-R = anorexia nervosa restricting subtype; AN-BP = anorexia nervosa binge purge subtype. BMI = body mass index; EDE = eating disorder examination; CIA = clinical impairment assessment. The descriptive statistics for AN subtype are presented as frequency (percentage)

#### Set shifting

Of the 120 participants, 37 (31%) had both baseline and end of treatment data, 28 (23%) were missing both baseline and end of treatment data, 3 were missing only baseline data, and 52 were missing only end of treatment data. As shown in Table 1, no baseline variables predicted whether set shifting data were missing at baseline or end of treatment. Therefore, we can conclude that both baseline and end of treatment set shifting data were missing at random, and analyses contained up to 89 (74%) of the study participants.

### Aim 1: executive functioning as an outcome

#### Central coherence

**Fig. 1:**
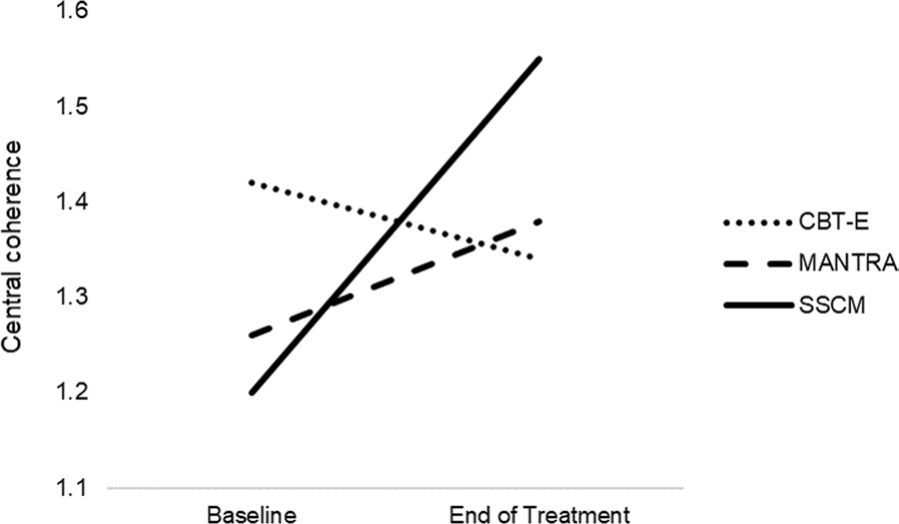
Mean central coherence by time (baseline, end of treatment) and group (CBT-E, MANTRA, SSCM)

There was a significant main effect of time, *F* (1, 38) = 4.76, *p* = 0.04, indicating that central coherence improved from baseline (*M* = 1.30, *SD* = 0.31) to end of treatment (*M* = 1.42, *SD* = 0.34). The effect size, adjusted for correlated data [37], showed that this was a small-to-medium effect (-0.32, 95% CI = -0.11 to -0.76). While there was no main effect of group, *F* (2, 38) = 0.20, *p* = 0.82, there was a significant interaction between time and group, *F* (2, 38) = 4.86, *p* = 0.01. As shown in Fig. 1, this interaction indicated that over time central coherence decreased in CBT-E but improved in MANTRA and SSCM. The rate of improvement over time was greatest in SSCM.

#### Set shifting

There was a significant main effect of time, *F* (1, 34) = 9.08, *p* = 0.005, indicating that set shifting improved from baseline (*M* = 9.58, *SD* = 7.42) to end of treatment (*M* = 5.91, *SD* = 2.25). The adjusted effect size showed that this was a small-to-medium effect, -0.37, 95% CI = -0.83 to -0.09. There was no main effect of group, *F* (2, 34) = 0.18, *p* = 0.84, nor interaction between time and group, *F* (2, 34) = 0.89, *p* = 0.42.

### Aim 2: executive functioning as a moderator of treatment outcome

While baseline set shifting did not moderate treatment outcome, there was a significant interaction between baseline central coherence and time for all treatment outcome variables. With the exception of BMI, the interaction indicated that participants with low baseline central coherence improved more from baseline to 12-month follow-up than those with high baseline central coherence. More specifically, the adjusted within-group effect sizes showed that participants with low baseline central coherence had a greater decrease in eating disorder psychopathology (-0.97, 95% CI = − 1.64 to − 0.29) and clinical impairment (− 1.39, 95% CI = − 2.12 to  −0.66) than those with high baseline central coherence (respective effect sizes of − 0.65, 95% CI = − 1.29 to − 0.02 and − 0.41, 95% CI = − 1.06 to 0.23). Although BMI increased more among participants with high baseline central coherence (0.71, 95% CI: 0.04 to 1.38) than those with low baseline central coherence (0.64, 95% CI: − 0.04 to 1.32), this was not clinically significant as both effects were moderate-to-large. Table 2 provides the descriptive statistics and Table 3 the inferential statistics.

**Table 2:** Means (standard deviations) for treatment outcomes by time and executive functioning (low, high)

	Baseline	Mid-treatment	End of treatment	6-month follow-up	12-month follow-up
**Central coherence**					
BMI					
Low	16.58 (1.18)	18.12 (1.90)	18.81 (2.86)	19.27 (2.99)	18.96 (2.48)
High	16.87 (1.05)	17.63 (1.44)	18.34 (1.56)	18.35 (1.67)	18.72 (2.00)
EDE					
Low	3.63 (1.27)	2.31 (1.29)	1.66 (1.21)	1.78 (1.50)	1.42 (1.11)
High	3.32 (1.33)	2.38 (1.15)	1.73 (1.41)	1.85 (1.42)	1.54 (1.40)
CIA					
Low	34.55 (9.51)	23.69 (14.12)	15.76 (12.47)	17.81 (14.30)	21.13 (14.11)
High	33.71 (11.81)	26.56 (10.58)	19.61 (12.24)	20.38 (14.84)	18.53 (17.27)
**Set shifting**					
BMI					
Low	16.39 (1.19)	17.97 (1.94)	19.05 (2.41)	19.15 (2.87)	19.25 (2.44)
High	17.10 (0.97)	18.21 (1.46)	18.84 (2.48)	18.95 (2.27)	18.84 (2.55)
EDE					
Low	3.68 (1.21)	2.27 (1.05)	1.50 (1.21)	1.73 (1.38)	1.31 (1.11)
High	3.32 (1.24)	2.47 (1.33)	2.01 (1.20)	2.09 (1.28)	1.81 (1.23)
CIA					
Low	34.64 (9.64)	24.00 (12.35)	15.63 (11.74)	17.83 (14.90)	18.58 (14.69)
High	32.95 (10.75)	25.13 (13.74)	22.19 (14.34)	23.18 (15.38)	23.65 (16.60)

BMI = body mass index; EDE = eating disorder examination; CIA = clinical impairment assessment

**Table 3:** Linear mixed model analyses showing baseline central coherence moderated change over time in treatment outcome variables

	Main effects *F* (df), *p*		Interaction *F* (df), *p*
	Time	Baseline executive functioning	Time x baseline executive functioning
**Central coherence (n = 41)**			
BMI	13.32 (4, 10.92), < 0.001	0.99 (31, 9.00), 0.55	**3.89 (133, 8.27), 0.02**
EDE	91.28 (4, 7.60), < 0.001	1.27 (31, 8.43), 0.38	**4.98 (115, 7.60), 0.01**
CIA	9.90 (4, 7.18), 0.005	1.53 (31, 6.61), 0.30	**10.99 (97, 6.67), 0.004**
**Set shifting** **(n = 37)**			
BMI	6.30 (4, 21.23), 0.002	0.67 (14, 21.94), 0.78	1.60 (55, 20.59), .12
EDE	23.48 (4, 22.57), < 0.001	0.65 (14, 21.56), 0.80	1.32 (55, 21.01), .25
CIA	20.91 (4, 13.33), < 0.001	0.75 (14, 21.00), 0.70	2.24 (51, 11.31), 0.07

BMI = body mass index; EDE = eating disorder examination; CIA = clinical impairment assessment; n = the number of cases included in the linear mixed model analyses

Bold inidcates significant interaction between baseline central coherence and time for all treatment outcome variables

### Aim 3 executive functioning as a predictor of early change and remission

Neither baseline central coherence nor set shifting predicted early change or remission at end of treatment, 6-month follow-up, or 12-month follow-up. Table 4 displays the descriptive and inferential statistics.

**Table 4:** Logistic regression analyses predicting early change and remission from baseline executive functioning, and means (standard deviations) for baseline executive functioning for early change versus no early change and remission versus no remission

	*M* (*SD*) baseline		OR (95% CI)
	Early change	No early change	
Central coherence (n = 41)	1.27 (0.34)	1.38 (0.23)	2.93 (0.18, 47.31)
Set shifting (n = 37)	9.13 (6.87)	11.71 (9.23)	1.04 (0.94, 1.15)

	*M* (*SD*) baseline		OR (95% CI)
	Remission	No remission	
**Central coherence**			
End of treatment (n = 41)	1.33 (0.29)	1.27 (0.34)	1.70 (0.21, 13.44), 0.62
6-month follow-up (n = 41)	1.22 (0.30)	1.32 (0.33)	0.38 (0.05, 3.28), 0.38
12-month follow-up (n = 41)	1.27 (0.29)	1.30 (0.35)	0.76 (0.10, 5.54), 0.79
**Set shifting**			
End of treatment (n = 37)	11.15 (8.16)	8.79 (6.82)	1.05 (0.95, 1.15), 0.36
6-month follow-up (n = 37	13.11 (8.85)	8.50 (6.51)	1.08 (0.98, 1.19), 0.13
12-month follow-up (n = 36)	11.00 (7.76)	8.82 (7.21)	1.04 (0.95, 1.14), 0.39

*Notes*. *M* = mean; *SD* = standard deviation; OR = odds ratio; CI = confidence interval; n = the number of cases included in the logistic regression analyses

## Discussion

We conducted exploratory, secondary data analyses to investigate whether executive functioning can change over the course of treatment, determine whether baseline executive functioning moderated treatment outcomes, and examine whether baseline executive functioning predicted early change or remission. While participants were a subset of those from a previous investigation [4], we believe this to be a representative sample as central coherence and set shifting data were missing at random with one exception—those with higher eating disorder psychopathology at baseline were less likely to have missing baseline central coherence data. This may reflect the fact that the data came from a treatment study and those with more severe eating disorder symptoms were attending all treatment/assessment sessions as they needed the support. This can be viewed as a strength as our results may extrapolate to those with more severe symptomatology.

### Executive functioning as an outcome

In line with research showing that executive functioning can change over treatment [13], we found that set shifting clearly improved from baseline to end of treatment. The results for central coherence were less clear cut, with improvements only noted in MANTRA and SSCM. Given that MANTRA explicitly targets thinking styles, we might expect to see central coherence most improved in this group. Rather, we found that central coherence improved more over treatment in SSCM. As mentioned, a core element of SSCM is supportive psychotherapy with up to half of each session focused on content dictated by the client [14–16]. It is possible that having to think through everything that is going on in one's life and prioritise which topics, issues, or concerns are most important to discuss in session each week may promote bigger picture thinking. Moreover, focusing on content that is most salient to the individual may enable them to work through the specific stuck points that are consuming their attention and getting in the way of viewing their challenges and life in a big picture way.

### Executive functioning as a moderator

We found that people with low baseline central coherence had a greater decrease in eating disorder psychopathology and clinical impairment from baseline to 12-month follow-up than those with high baseline central coherence. A possible explanation for this finding is that the big picture of recovery may seem daunting and interfere with treatment progress, whereas an ability to focus on the details of changes that need to happen each week (e.g., changes in eating step by step) is what is needed and helpful for more rapid improvement. This finding supports the proposition that the detail-focused thinking style commonly observed among people with anorexia nervosa can be both a vulnerability and a strength. More specifically, while thinking in terms of details, for example, about food and weight could pose a vulnerability for the development and maintenance of anorexia nervosa, it could also break down the process of recovery into smaller, less overwhelming, and more achievable steps.

### Executive functioning as a predictor

We found that baseline central coherence and set shifting did not predict early change or remission. This suggests that other variables may be more important or influential than baseline executive functioning. For example, baseline variables such as BMI, motivation, eating disorder psychopathology, depression diagnosis, self-esteem, and anorexia nervosa subtype have been shown to predict how well people with anorexia nervosa do in treatment [38–40].

### Limitations

The present study had several limitations. First, participants were primarily white females. Thus, results are unable to be extrapolated to a more diverse sample of people with anorexia nervosa. Second, like many randomised controlled trials, strict exclusion criteria were applied (e.g., severe physical or mental illness such that outpatient treatment was inappropriate, current severe substance dependence, and current use of atypical antipsychotics because of the weight gain properties of these drugs). This means that the sample may not be representative of all people presenting for outpatient treatment of anorexia nervosa. Third, the substantial amount of missing executive functioning data limited our power to investigate moderation. It also may have introduced bias, but overall, the subset of participants included in our analyses were representative of the sample as a whole. Finally, results and *p* values should be interpreted with some caution due to the small sample size and exploratory nature of the study.

### Conclusion

Our findings highlight several directions for future research. First, replication of our results is required before definitive conclusions can be made about whether set shifting can improve over treatment and whether baseline central coherence moderates treatment outcomes. Second, given the inconsistent findings regarding whether executive functioning can improve over treatment, it would be beneficial for future treatment studies to routinely assess executive functioning. Third, a recent meta-analysis by Keegan et al. [5] found that people with non-underweight eating disorders, such as binge eating disorder and bulimia nervosa, have central coherence and set shifting inefficiencies that are comparable to those observed among people with anorexia nervosa. Therefore, it would be of interest to examine whether the same pattern of results is obtained in the non-underweight group. Finally, and most importantly, our findings suggest some hypotheses for further testing in an adequately powered study. Specifically, while a detail-focused thinking pattern can pose a vulnerability for the development and maintenance of anorexia nervosa, it also offers a pathway to a focused drive and determination that can be weaponised against the eating disorder when directed towards recovery.

## Supplementary Information

**Additional file 1**. **Supplementary Table 1.** Basic demographic information for participants who had both baseline and end of treatment central coherence and set shifting data.

## Data Availability

The data that support the findings of this study are available from the corresponding author upon reasonable request.
