# Supplementary material for: An exploratory examination of executive functioning as an outcome, moderator, and predictor in outpatient treatment for adults with anorexia nervosa
Source: J Eat Disord. 2022 Jun 17;10:83. doi: 10.1186/s40337-022-00602-0 (PMC9206373; doi:10.1186/s40337-022-00602-0)
Supplement: Supplementary file 1 — Additional file 1. Supplementary Table 1. Basic demographic information for participants who had both baseline and end of treatment central coherence and set shifting data. [file 40337_2022_602_MOESM1_ESM.docx]

**Supplementary Table 1**

*Basic demographic information for participants who had both baseline and end of treatment central coherence and set shifting data*

| Demographic variable | Central coherence (n = 41) | Set shifting (n = 37) |
| --- | --- | --- |
| Gender | 97.6 | 97.3 |
| Age | 24.24 (9.86) | 25.41 (10.40) |
| Baseline BMI | 16.72 (1.11) | 16.77 (1.12) |

*Notes.* Gender = percentage female. Age = mean (standard deviation) age in years. Baseline BMI = mean (standard deviation).
